# Rate of hospitalizations and underlying reasons among people with Parkinson’s disease– Population-based cohort study in UK primary care

**DOI:** 10.3233/JPD-212874

**Published:** 2022-01-01

**Authors:** Olaitan Okunoye, Laura Horsfall, Louise Marston, Kate Walters, Anette Schrag

**Affiliations:** a Department of Clinical and Movement Neurosciences, University College London, UK; b Department of Primary Care and Population Health, University College London, UK

**Keywords:** Parkinson’s disease, Hospitalization, Incidence, Reasons, Primary care

## Abstract

**Introduction:**

Hospitalization in Parkinson’s disease (PD) is associated with reduced quality of life, caregiver burden and high costs. However, no large-scale studies of rate and causes of hospitalizations in patients with PD have been published.

**Objective:**

To investigate the rate and reasons for hospitalization and factors associated with hospitalization among people with PD compared to the general population.

**Methods:**

We examined rate and causes of admission in PD patients and matched controls in The Health Improvement Network from 2006 to 2016. Multivariable Poisson regression was used to explore the effects of age, gender, social deprivation, urbanicity and practice geographic location on hospitalization.

**Results:**

In longitudinal data from 9,998 newly diagnosed individuals with PD and 55,554 controls without PD aged ≥50years, 39% of PD patients and 28% of controls were hospitalised over a median follow-up 5.1years. The adjusted incidence rate ratio(IRR) of hospitalization in PD compared to controls was 1.33(95%CI:1.29-1.37) and rose with increased follow-up duration. Hospitalization rate was overall higher in the older age groups, but the adjusted IRR of hospitalization compared to controls was highest in the youngest age group. PD patients were more often admitted with falls/fractures, infections, gastrointestinal complications, PD, dementia, psychosis/hallucinations, postural hypotension, electrolyte disturbances, stroke and surgical procedures and slightly less often due to hypertension.

**Conclusion:**

People with PD have an increased hospitalization rate compared to controls, particularly in the younger age groups, and it increases with longer disease duration. The complications of motor and non-motor features of PD are amongst the main reasons for admission, some of which could be managed preventatively to avoid admissions.

## Introduction

Hospitalizations are associated with worse outcomes in people with Parkinson’s disease (PD) and may lead to worsening of their disease [1]. They can lead to worsening of motor symptoms, hospital-acquired infections, medication errors and higher mortality following surgical procedures [2–5]. Hospital stay in patients with PD is longer than in other patients [6] and re-admissions are increased [7]. In addition, the cost of hospitalization is high and likely to increase with increasing adult population [6]. There are few studies [8, 9] on rate of hospitalization of people with PD. Identifying rates and causes of hospitalization could help plan for and reduce unwarranted admissions, morbidity and financial burden associated with PD [6]. We therefore investigated the rate of and reasons for hospitalization among people with PD in the UK compared to people without PD using a large primary care database.

## Methods

### Source of data

We conducted a cohort study from 1^st^ of January 2006 until 31^st^ of December 2016 using electronic primary healthcare records from IQVIA™ Medical Research Data (IMRD) that incorporates data supplied by The Health Improvement Network(THIN), a propriety database of Cegadim SA which currently covers about 6% of the UK population and contains longitudinal data on over 17 million people [10]. Available computerized data include demographics, details of GP consultation, diagnoses from referrals from specialists and hospital admissions, drug prescriptions, laboratory tests results and other health indicators including blood pressure and smoking status. The Read classification, a hierarchical coding system is used to code individual diagnoses, findings of examination and hospital attendances data [11].

### Study Design

The study population comprised of individuals who were 50 years old and more and were actively registered for at least six months within the general practice from 1^st^ January 2006 to 31^st^ December 2016 (n=3,195,391). We conducted a cohort study using data from THIN, among adults with incident PD compared with up to six people without PD matched by age, gender, calendar year and practice. Individuals entered the study at the latest date of registration with a general practice plus six months[12], 50^th^ birthyear, or after the practice met the quality assurance criteria of acceptable computer usage (ACU) [13] and acceptable mortality reporting (AMR) [14] and individuals in the comparison cohort entered the study on a matched date (index date). The index date was taken as the earliest date of PD diagnosis Read code entry or antiparkinsonian drug code entry.

Study participants were then followed up until they died, transferred out of the practice, the practice stopped contributing data to THIN or end of follow-up, whichever was earliest. The National Health Service South-East Multicentre Research Ethics Committee gave approval for the use of THIN in 2003. This study was approved by IQVIA Medical Research Scientific Review Committee in June 2019 (SRC Reference Number: 19THIN034).

### Parkinson’s disease case ascertainment in THIN

Using the Read classification [11], PD was defined by a diagnosis Read code and at least two prescriptions of any of the five major antiparkinsonian medications (Levodopa-containing medications, Dopamine-receptor agonists, Amantadine, Monoamine-oxidase--B inhibitors-rasagiline and selegiline and Catechol-O-methyl transferase inhibitors (entacapone and tolcapone)). People with a first ever recording of PD diagnosis were identified through a computer search using Read code lists which were generated through previously published methods [15]. This PD case ascertainment has been reported to have a 90% validity in another similar database: General Practise Research Database (GPRD) [16].

### Exclusion criteria

Prior to study entry, all individuals with a history of PD were excluded. Those with restless leg syndrome without PD who had been treated with dopamine agonists were also excluded. In addition to individuals with less than six months of data from registration within a general practice, we also excluded those with a diagnosis in the first six months after registration because they are likely to represent previous medical records rather than true new entries of PD diagnoses [12].

### Study outcomes

The main outcome of interest was hospital admissions which were identified through Read codes for admission. Discharge Read codes were used where the admission codes were not available with the assumption that an admission preceded the discharge. Admission/discharge codes within 21 days of a previous admission/discharge code were considered same admission as the average length of stay in hospital in the available literature was 17 (SD 7) days.

Diagnoses associated with admissions were identified using Read code entries from date of diagnosis up to 28 days following discharge (allowing time for discharge summaries to get to the GP practice from secondary care) after a hospital admission. Read code lists for admission/discharge and those for reasons for admission were developed using published methods [15]. A combination of Read codes and blood pressure readings were used to define those with hypertension.

### Covariates

Data on demographics and social deprivation were collected for each individual in our cohort. Social deprivation was measured by Townsend score. This is categorised into quintiles from 1 (least deprived) to 5 (most deprived). Information on urban-rural living is provided through the Government classification system. The boundaries of the former strategic health authorities based in England are linked to UK geographic regions.

### Statistical analysis

Baseline characteristics of the study cohort were compared using chi-squared test. Incidence rates of hospitalization per 1,000 person-years with 95%CI were calculated. Data were split by one-year intervals and calendar year was used as a continuous variable with age taken as the timescale. Age categories were created in ten years intervals. Multivariable Poisson regression analyses were used to estimate incidence rate ratios and marginal effects adjusted for age, calendar year and other covariates. The marginal effect which is an estimation of how much the incidence rate is predicted to change for every unit of change in an exposure variable is useful for visualizing effects of interactions that are difficult to interpret directly from the model coefficients. The marginal effects for fixed values of calendar year were calculated while holding all other variables in the model at their observed values and estimating standard errors by using the delta method. Incidence rate ratios (after the regression analyses) for the categorical variables were computed. We used the Wald test to calculate p-values for multiplicative interaction terms and categorical variables. In order to estimate robust standard errors, we used practice identifiers to account for the effects of clustering of observations within general practices. We investigated overdispersion by running and comparing the outputs of negative binomial models. All statistical analyses were conducted using Stata version 16MP (Stata Corporation, College Station, Texas).

## Results

### General characteristics

The study cohort included 9,998 people with PD and 55,554 matched on age, calendar year, gender and general practice without PD. Mean age of the study population was 74 years (SD 8.18). Our PD cohort had more males (60.8% males vs 39.3% females). Median follow-up period was 5.10 years (IQR 4.60 to 5.80).

A total of 56,391 of hospital admissions were identified, 12,452 in the PD cohort and 43,939 in the non-PD cohort during the study period. These admissions were from 19,598 individuals: 3,857 people (39% of those with PD) were from the PD cohort and 15,741 people (28% of those without PD) were from the non-PD cohort. The median number of admissions among those who were admitted was 2 for both groups. The mean number of admissions among those admitted for the PD cohort was 3 (SD 5) and for the non-PD control cohort was 2 (SD 3) (Table 1 and Table 2).

Of the PD cohort, 1,526 had one record of admission and 2,331 had two or more records of admissions. Females (61%), those aged 70 to 79 years (63%) and those from the least deprived areas (61%) had two or more admissions (p <0.001) (Supplemental Table 1).

Potential reasons for admission were identified in 3,493 of the 3,857 PD patients, and 11,746 of the 15,741 non-PD cohort (Table 3).

### Incidence rate of Hospital admissions

The overall incidence rate of hospital admissions among people with PD was 146.15 per 1,000 person-years (95%CI: 141.61 to 150.84) and 108.98 per 1,000 person-years (95%CI: 107.29 to 110.70) in those without PD (Supplemental Table 2).

**Fig. 1:**
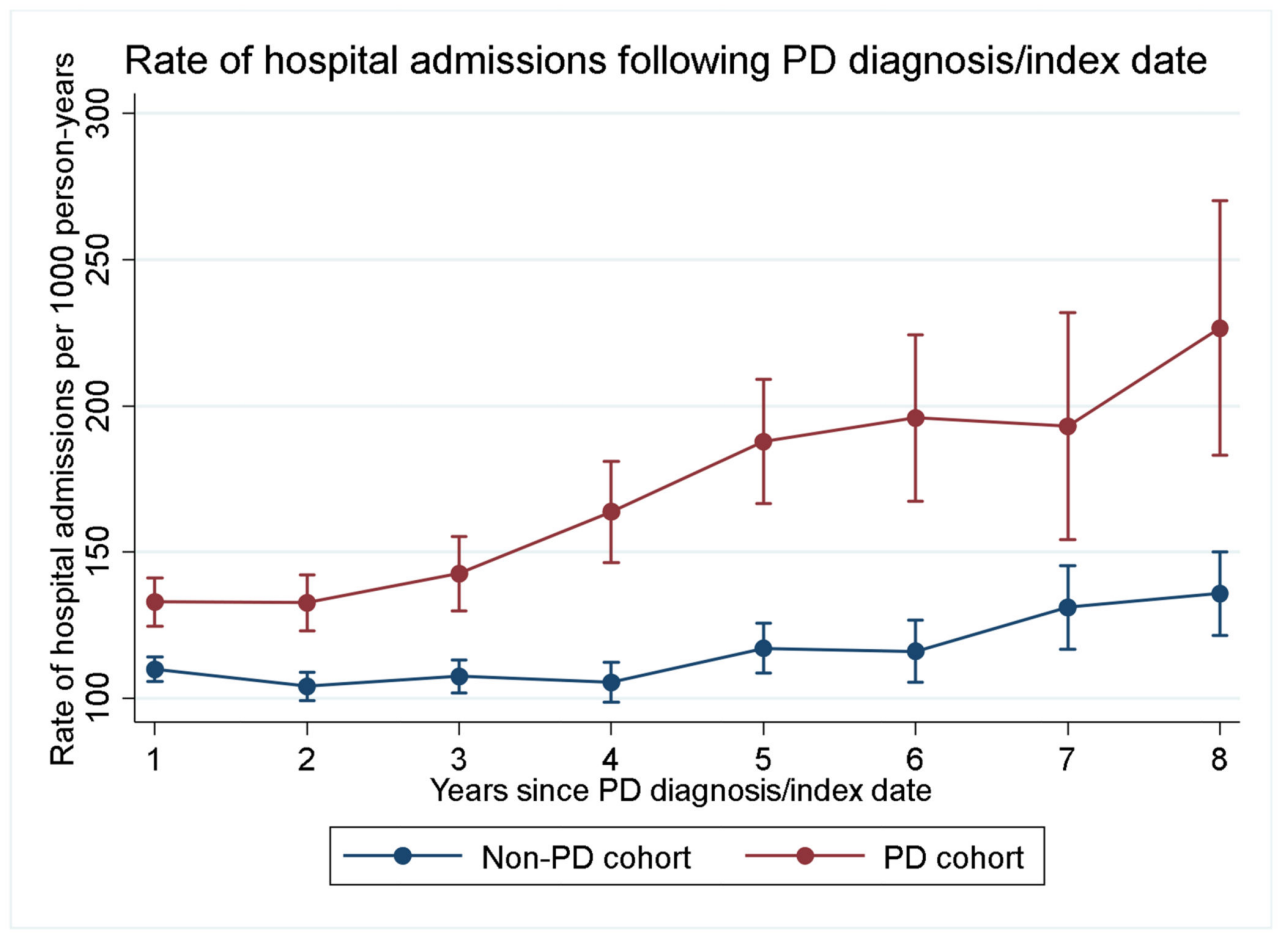
Association between disease duration and incidence rate of hospital admissions for people with Parkinson’s disease and the non-Parkinson’s disease control cohort.

After accounting for age, gender, calendar year, geographic location of practice, urbanization, socio-economic and smoking status, incidence of hospital admissions among individuals with PD was higher than the non-PD cohort (IRR: 1.33, 95%CI:1.29 to 1.37) (Table 2). Rate of hospital admissions increased with longer time from diagnosis or index date (Figure 1 and Supplemental Table 3).

Within the PD cohort, overall incidence rate stratified be gender was 147.48 (95% CI: 141.63 to 153.58) for males and 144.14 (137.06 to 151.58) for females.

After adjusting for age, calender year, geographic location of practice, urbanization, socio-economic and smoking status there were no sex related differences in hospitalisation (Supplementary Table 4 and 6).

### Factors associated with increased hospital admissions

**Fig. 2:**
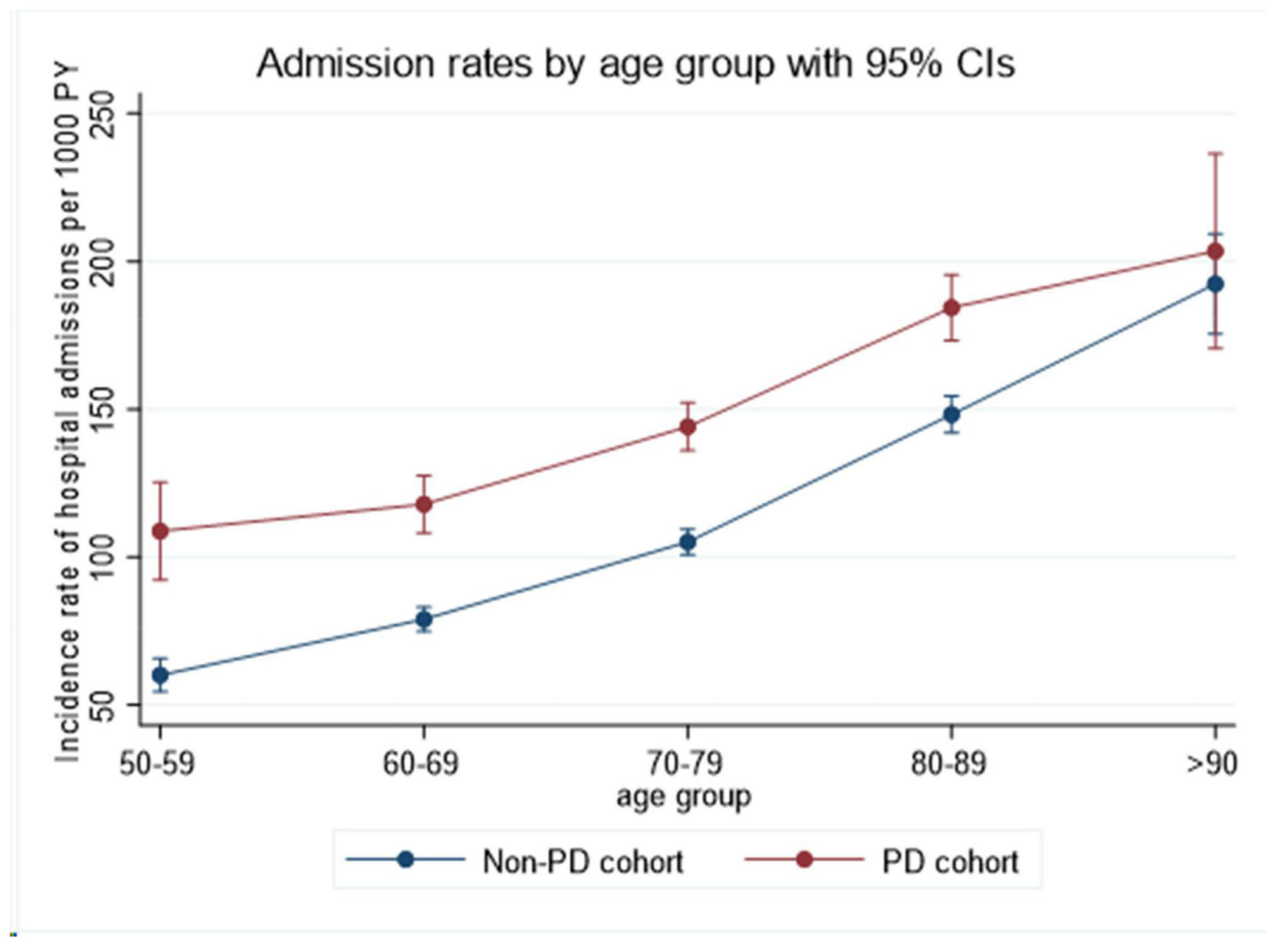
Association between age group and incidence rate of hospital admissions for people with Parkinson’s disease and the non-Parkinson’s disease control cohort.

Among the PD cohort, after accounting for age, gender, calendar year, social deprivation, smoking, urbanicity and practice geographical location, adjusted incidence rates for admissions in the younger age groups (50 to 59 and 60 to 69) were approximately 40% higher than those in the non-PD cohort in the same age groups. Adjusted incidence rates for admissions appeared to gradually level up in the older age groups: the adjusted incidence rate for age group 50 to 59 in the PD versus non-PD cohort was 108.95 per 1,000 person-years (95%CI 92.39 to 125.50) versus 60.28 per 1,000 person-years (95%CI: 54.70 to 65.86) whereas the adjusted incidence rates for age group >90 years for the PD versus non-PD cohort was 204.09 per 1,000 person-years (95%CI: 171.07 to 237.11) versus 192.59 per 1,000 person-years (95%CI: 175.60 to 209.57) (Figure 2 and Supplemental Table 5).

There was no relationship between rates of hospitalization between people with and without PD regarding urbanicity, UK countries and social deprivation (Supplemental Table 5).

### Reasons for admissions

Whilst the most common potential reasons for hospitalization in both groups were falls, fractures, infections (mainly chest, urinary tract and skin and subcutaneous tissue infections), gastrointestinal complications (mainly dysphagia, constipation, nausea and vomiting) and dementia, they were more common in patients with PD, as were postural hypotension, electrolyte disturbances, stroke, surgical procedures (such as laser surgery for glaucoma, neurosurgery, cardiothoracic, plastic and gastrointestinal surgery in addition to other non-specified minor and major surgical procedures) and psychosis/hallucinations, the latter being 7 times more common in the PD population. Additionally, patients with PD were admitted due to their PD only (i.e the potential reason for admission recorded was Parkinson’s disease). On the other hand, hypertension was slightly less common reason for admission in those with PD than controls. There were no differences in the rates of admissions for myocardial infarction/ischaemic heart disease, congestive heart failure and other cardiovascular causes and cancer between people with PD and controls (Table 3).

### Reasons for admission stratified by age group

Since younger patients may have different reasons for admissions, we stratified reasons for admission by age groups: 50 to 69 years and 70 years and more.

Stroke and postural hypotension were a more common reason for admission in those with PD than in matched controls only in in the older age group and electrolyte imbalance only in the younger PD population. Only in the older PD population were myocardial infarction/ischaemic heart disease, congestive heart failure and hypertension less common than in matched controls. Other differences between PD and controls were seen in both age groups (Supplemental Table 7 and 8).

### Reasons for admission among the PD cohort stratified by gender

Within the PD cohort, reasons for admission were stratified based on gender. Myocardial infarction/ischaemic heart disease and postural hypotension were a more common reason for admissions among men with PD. Whilst falls were a slightly more common reason in men with PD, fractures were a considerably more common reason for admission in females with PD. Other reasons for admissions were seen equally in both male and female patients with PD (Supplemental Table 9).

## Discussion

### Rate of Hospital admissions

The rate of hospital admissions among people with PD in UK primary care was 1.33 times higher than the matched control population and was increased from the year of diagnosis but rose with increasing disease duration. Whilst there are no comparable studies in the UK population, this rate ratio of hospitalization is comparable, albeit slightly lower, to the results of previous studies where people with Parkinsonism were reported to be 1.5 (USA) [8] and 1.44 (Canada) [9] times more likely to be admitted to hospital compared to controls. Our slightly lower rates could be explained by the fact that these studies were conducted in prevalent cohorts rather than incident cohorts which, whilst not reported, are likely to have had longer average disease durations. In addition, they included patients with parkinsonism, including atypical parkinsonism which has a higher morbidity and worse prognosis. Furthermore, differences in healthcare systems, for example predominantly out-patient based management and lower number of hospital beds per population in the UK, could have contributed to the lower rate ratio for hospitalization of people with PD in the UK.

The difference in admission rates was most marked in the younger age groups of people with PD but gradually levelled off in the older age groups. Nevertheless, patients in the older age group were still being admitted more frequently than patients with PD in the younger age group. There were no differences in rates of admission between males and females in people with and without PD, and no associations between rates of hospital admissions between people with and without PD regarding urbanicity, UK countries and social deprivation.

### Reasons for hospitalization

Potential reasons for hospitalization identified in our study is in keeping with the known features of advancing PD, including its increasing motor complications with freezing, motor fluctuations and postural instability, and a range of non-motor symptoms such as neuropsychiatric complications, postural hypotension, constipation, urinary dysfunction and complications of treatment, including electrolyte disturbances, hallucinations and dyskinesias [17]. Deteriorating motor control may lead to admissions either due to deterioration of mobility or the consequences of falls and fractures [18], dysphagia leading to aspiration pneumonia [19], bladder dysfunction leading to urinary tract infections, constipation necessitating admission, and antiparkinsonian medication together with PD contributing to postural hypotension [20, 21] and electrolyte disturbances and exacerbating delusions particularly in those with dementia. Whilst some of these are not necessarily preventable, careful monitoring for infections, falls risk assessments, assessment of bladder residual and rapid testing for urinary tract infections, adjustment and review of medications, swallowing assessments and multi-disciplinary treatment such as speech therapy and physiotherapy [22], all have the potential to reduce the risk of hospitalization [23].

Of note, hypertension was slightly less common reason for admission in those with PD than in controls, which may be a reflection of the reduction of blood pressure by PD and antiparkinsonian medications. Similarly, in the older age group, myocardial infarction/ischaemic heart disease and congestive heart failure was a less common diagnosis at admission in the PD cohort. On the other hand, stroke was a more common reason for admission in people with PD in the older age group, even after adjustment for smoking status. This has not been reported previously but it could be speculated that blood pressure variability with hypotension and nocturnal supine hypertension may increase the risk of stroke [24]. This finding highlights the importance of carefully managing complication of postural hypotension, which often significantly affects quality of life but is typically difficult to manage and may result in supine hypertension. On the other hand, in the younger age group, admission due to myocardial infarction/ischaemic heart disease and congestive heart failure, were not different between patients with PD and matched controls, perhaps reflective of the lower rate of hypertension.

Similar to our study, several studies [8, 9, 25] have reported infections, falls and fractures as the main reasons for hospital admissions among people with PD. Whilst also common in the older population without PD [26], it was higher in the PD population in both older and younger age groups. Psychosis/hallucinations were considerably more common reasons for admission in patients with PD than in the non-PD population, and this difference was most marked in the younger age group, with a 19-fold increase in risk of admission compared to controls. Dementia was also a more common cause of admission in the younger age group, with a 11-fold higher rate compared to controls and is known to be associated with psychosis in PD.

Whilst we did not examine medication doses, antiparkinsonian medication doses are typically higher in the younger age group which is likely to have contributed to the higher rate of hallucinations and psychosis [27–29] as well as to the higher rate of postural hypotension as a more common cause of admission in the younger age group of patients with PD.

Previous reports on the rates of admission for cardiovascular diseases and stroke in patients with and without PD have provided mixed results [8, 20, 21]. Some studies [8] showed that people with PD compared to controls without PD are more likely to be admitted for cardiovascular diseases such as myocardial infarction/ischaemic heart disease, chronic heart failure and stroke while others found no difference or less representation [20, 21, 30]. Overall, there is speculation that the risk of cardiovascular diseases and stroke is low in people with PD. This is attributed to reduced vascular risk factors and lower smoking rates in people with PD. In contrast, in a recent meta-analysis, the risk of cardiovascular disease and stroke were reported to be higher in PD especially in the older age group [31], similar to this study.

In keeping with previous studies [21], reason for admission attributed to cancer was not different between patients with PD and matched controls. This was same for both PD age groups. The risk of cancer has previously been reported to be lower in PD compared to control populations [32, 33].

In this study, we show that people with PD were more likely to be admitted for gastrointestinal complications including dysphagia, nausea and vomiting, and constipation compared to the control cohort with higher rates. Similar to our study, one study [21] reported gastrointestinal complications as a top reason for hospitalization in PD. Gastrointestinal problems such as dysphagia may lead to swallowing problems resulting in aspiration pneumonia which is reported to be the commonest cause of death in people with PD [34]. Early identification of dysphagia through regular assessment of swallowing function and implementation of changes to dietary consistency may prevent aspiration pneumonia [9] and subsequent hospitalization.

### Strengths and limitations

In addition to our large cohort size, a strength of this study is that we examined incident cases which allows follow up from early disease, and is more informative on an individual prognostic basis. The main limitation of this study is that we were unable to validate hospital admissions or reasons for hospitalization in the database since these data were based on primary care recording of secondary care reports, which may have led to underreporting of admissions and diagnoses. Attribution of symptom recording in relation to admission may also not be precise as coinciding admission date and symptom recording does not necessarily indicate they are directly related. For some admissions, the diagnosis associated with admission could not be determined. Another limitation is that we were unable to account for the presence/absence of caregivers which may contribute or modify risk for hospital admissions, the type of treatment received, and disease severity because this is not well recorded in this database.

Finally, we relied on GP codes for clinical diagnoses of PD which may have resulted in misclassification of PD cases and inclusion of some atypical cases.

Nevertheless, this is by far the largest study of hospital admissions in PD conducted to date, in a representative sample and based on routine records, avoiding recall and selection bias. These data therefore provide reasonably robust information on the rate and diagnoses associated with admissions of patients with a diagnosis of PD.

## Conclusion

Our study found higher rates of hospitalization in people with PD, due to a range of causes many of which relate to symptoms or complications of PD. It is important to develop strong evidence based specific interventions directed at specific complications of PD in order to reduce the risk of hospitalization among people with PD resulting in a better quality of life for the patients, reduced burden and costs.

## Supplementary Material

Supplemental materials

## Figures and Tables

**Table 1:** Cohort characteristics

Characteristics	PD cohort (*n* = 9,998)	Non-PD control group (*n* = 55,554)	*p-value
**Gender *n (%)***			
Men	6,093 (60.94)	33,778 (60.68)	0.624
Women	3,905 (39.06)	21,886 (39.32)	
**Age group *n (%)***			
50 to 59 years	786 (7.86)	4,403 (7.91)	0.787
60 to 69 years	2,320 (23.21)	12,961 (23.29)	
70 to 79 years	4,149 (41.50)	23,170 (41.63)	
80 to 89 years	2,507 (25.08)	13,941 (25.05)	
90 years and over	236 (2.35)	1,189 (2.31)	
**Townsend score *n (%)***			
1(least deprived)	2,791 (27.92)	13,746 (24.69)	<0.001
2	2,300 (23.00)	12,399 (22.27)	
3	1,872 (18.72)	10,815 (19.43)	
4	1,421(14.21)	8,728 (15.68)	
5(most deprived)	882 (8.82)	5,565 (10.00)	
Missing	732 (7.32)	4,411 (7.92)	
**Urbanicity *n (%)***			
Urban	5,785 (57.86)	31,878 (57.27)	<0.201
Town	1,024 (10.24)	5,632 (10.12)	
Rural	625 (6.25)	3,336 (5.99)	
No records	2,564 (25.65)	14,818 (26.62)	
**UK Countries *n (%)***			
England	38,837 (69.77)	7,006 (70.07)	0.918
Northern Ireland	2,804 (5.04)	492 (4.92)	
Wales	8,502 (15.27)	1,510 (15.10)	
Scotland	5,521 (9.92)	990 (9.90)	
**Smoking status *n (%)***			
Non-smoker	5,444 (54.45)	24,514 (44.04)	<0.001
Ex-smoker	3,091 (30.92)	18,624 (33.46)	
Current smoker	685 (6.85)	7,280 (13.06)	
Missing	778 (7.78)	5,259 (9.45)	

PD-Parkinson’s disease.

* Chi-squared test

**Table 2:** Number and rates of hospital admissions

Variables	PD cohort *n* = 9998	Non-PD control cohort *n* = 55,664	p-value
Number of people admitted at least once	3,857 (39%)	15,741 (28%)	<0.001
Median number of admissions among those admitted *n (IQR)*	2 (1 to 4)	2 (1 to 3)	
Person-years, per 1,000	26.39	144.43	
Unadjusted incidence rate, per 1,000 PY (95% CI)	146.15 (141.61 to 150.84)	108.98 (107.29 to 110.70)	
Unadjusted incidence rate ratio	1.34(1.30 to 1.39)	1 (reference)	<0.001
Incidence rate ratio (95% CI) adjusted age, gender, calendar year social deprivation, smoking, urban-rural and UK countries.	1.33 (1.28 to 1.37)	1 (reference)	<0.001

PD-Parkinson’s disease

**Table 3:** Reasons for hospitalization among the admitted cohort.

	PD cohort		Non-PD control cohort			
Reasons for hospital admission	Number admitted	Rate admitted for the reason per 1,000 person-years (95% Confidence Interval)	Number admitted	Rate admitted for the reason per 1,000 person-years (95% Confidence Interval)	Incidence rate ratio (95% Confidence Interval)	*p-value
Neuropsychiatric complications (psychosis and hallucinations)	138	5.23 (4.43 to 6.18)	97	0.67 (0.55 to 0.82)	7.59 (5.82 to 9.89)	<0.001
Dementia	282	15.73 (14.28 to 17.31)	668	4.50 (4.17 to 4.86)	3.49 (3.06 to 3.99)	<0.001
Myocardial infarction/Ischaemic heart disease	86	3.26 (2.64 to 4.03)	569	3.90 (3.59 to 4.23)	0.80 (0.64 to 1.01)	0.057
Congestive heart failure	95	3.60 (2.94 to 4.40)	649	4.48 (4.15 to 4.85)	0.80 (0.63 to 1.00)	0.052
Stroke	168	6.33 (5.44 to 7.36)	698	4.80 (4.46 to 5.18)	1.30 (1.11 to 1.52)	0.001
Hypertension	92	3.49 (2.84 to 4.28)	639	4.37 (4.04 to 4.72)	0.79 (0.63 to 1.00)	0.038
Gastrointestinal complications	417	15.80 (14.36 to 17.39)	1,330	9.21 (8.73 to 9.72)	1.69 (1.51 to 1.90)	<0.001
Falls	517	19.59 (17.97 to 21.35)	1,206	8.35 (7.89 to 8.83)	2.33 (2.10 to 2.59)	<0.001
Fractures	316	11.94 (10.69 to 13.33)	734	5.02 (4.67 to 5.41)	2.34 (2.03 to 2.69)	<0.001
Infections	444	16.82 (15.33 to 18.46)	1,623	11.23 (10.70 to 11.79)	1.50 (1.34 to 1.67)	<0.001
Other cardiovascular causes	212	8.00(6.99 to 9.15)	1,052	7.23 (6.81 to 7.69)	1.08 (0.93 to 1.26)	0.323
Cancer	250	9.47 (8.37 to 10.72)	1406	9.69 (9.20 to 10.21)	0.97 (0.85 to 1.11)	0.665
Postural hypotension	229	8.68 (7.62 to 9.88)	341	2.34 (2.10 to 2.60)	3.60 (3.05 to 4.26)	<0.001
Electrolyte imbalance	79	2.99 (2.40 to 3.73)	295	2.04 (1.82 to 2.29)	1.48 (1.15 to 1.90)	0.003
Parkinson’s disease	177	6.71 (5.79 to 7.77)	NA	NA	NA	NA
Surgical causes	273	10.31 (9.15 to 11.61)	1,107	7.66 (7.22 to 8.12)	1.30 (1.13 to 1.49)	<0.001
Not identified	82		3,327			

PD-Parkinson’s disease. NA-not applicable

* Mutually adjusted for age, gender, calendar year, social deprivation and smoking
