# Supplementary material for: Rate of hospitalizations and underlying reasons among people with Parkinson’s disease– Population-based cohort study in UK primary care
Source: J Parkinsons Dis. Author manuscript; Available in PMC 2022 Jan 26. (PMC7612266; doi:10.3233/JPD-212874)
Supplement: Supplemental materials [file EMS138301-supplement-Supplemental_materials.pdf]

Supplemental materials:

Supplemental Table 1: Cohort characteristics based on number of admissions among the PD population

**Characteristics**	**PD people with 1 record of admission**	**PD people with >1 record of admission**
**Number of PD people admitted** *n (%)*	1,526 (39.56)	2,331 (60.43)
**Gender** ***n (%)***		
Men	948 (40.46)	1,395 (59.54)
Women	578 (38.18)	936 (61.82)
**Age group** ***n (%)***		
50 to 59 years	126 (45.32)	152 (54.68)
60 to 69 years	345 (39.20)	535 (60.80)
70 to 79 years	626 (37.09)	1,062 (62.91)
80 to 89 years	389 (41.43)	550 (58.57)
90 years and over	40 (55.56)	32 (44.44)
**Townsend score** ***n (%)***		
1(least deprived)	426 (39.12)	663 (60.88)
2	378 (40.91)	546 (59.09)
3	271 (38.60)	431 (61.40)
4	215 (40.41)	317 (59.59)
5(most deprived)	144 (42.35)	196 (57.65)
No records	92 (34.07)	178 (65.93)
**Urbanicity** ***n (%)***		
Urban	852 (37.55)	1,417 (62.45)
Town	187 (44.95)	229 (55.05)
Rural	109 (43.78)	140 (56.22)
No records	378 (40.95)	545 (59.05)
**UK Countries** ***n (%)***		
England	1,037 (37.90)	1,699 (62.10)
Northern Ireland	80 (32.26)	168 (67.74)
Wales	229 (48.62)	242 (51.38)
Scotland	180 (44.78)	222 (55.22)
**Smoking status** ***n (%)***		
Non-smoker	808 (38.44)	1,294 (61.56)
Ex-smoker	488 (40.53)	716 (59.47)
Current smoker	116 (40.14)	173 (59.47)
Missing	114 (43.51)	148 (56.49)

PD-Parkinson's disease

Supplemental Table 2: Crude incidence rates of hospital admissions among people with PD and Non-PD control cohort

	Parkinson's disease cohort			Non-Parkinson's disease control cohort		
Variables	Events	Person-Years (1000)	Incidence rate (95% CI)	Events	Person-Years (1000)	Incidence rate (95% CI)
**Overall**	3,857	26.39	146.15 (141.61 to 150.84)	15,741	144.43	108.98 (107.29 to 110.70)
**Age group**						
50 to 59	183	1.70	107.35 (92.87 to 124.09)	574	9.49	60.51 (55.76 to 65.67)
60 to 69	692	5.95	116.38 (108.02 to 125.38)	2546	32.30	78.82 (75.82 to 81.94)
70 to 79	1570	11.03	142.35 (135.48 to 149.57)	6195	59.39	104.32 (101.75 to 106.95)
80 t0 89	1273	7.02	181.45 (171.75 to 191.70)	5680	39.22	144.83 (141.12 to 148.65)
>90	139	0.69	202.08 (171.13 to 238.63)	746	4.04	184.49 (171.71 to 198.21)
**Gender**						
Male	2343	15.89	147.48 (141.63 to 153.58)	9533	86.54	110.16 (107.97 to 112.39)
Female	1514	10.50	137.06 (137.06 to 151.58)	6208	57.89	107.23 (104.60 to 109.93)
Year						
2006	80	0.44	180.08 (144.64 to 224.20)	313	2.22	140.98 (126.20 to 157.50)
2007	211	1.20	175.76 (153.58 to 201.15)	752	5.91	127.32 (118.54 to 136.75)
2008	317	1.86	170.25 (152.50 to 190.06)	1196	8.74	136.81 (129.27 to 144.78)
2009	390	2.38	164.09 (148.59 to 181.21)	1459	11.03	132.26 (125.64 to 139.22)
2010	412	2.72	151.44 (137.50 to 166.79)	1571	12.97	121.12 (115.28 to 127.26)
2011	461	2.98	154.53 (141.05 to 169.30)	1706	15.24	111.94 (106.75 to 117.38)
2012	458	3.18	143.90 (131.30 to 157.70)	1973	17.35	113.74 (108.83 to 118.87)
2013	421	3.24	129.96 (118.12 to 142.99)	1914	18.66	102.56 (98.07 to 107.26)
2014	453	3.21	141.15 (128.73 to 154.76)	1923	19.47	98.76 (94.45 to 103.28)
2015	409	2.81	145.68 (132.22 to 160.50)	1606	17.59	91.33 (86.97 to 95.90)
2016	245	2.36	103.65 (91.45 to 117.47)	1328	15.26	87.03 (82.47 to 91.84)
**Townsend quintile**						
1 (least deprived)	1089	7.57	143.84 (135.55 to 152.65)	3938	37.03	106.34 (103.07 to 109.72)
2	924	6.16	150.0 (140.63 to 159.99)	3471	32.03	105.42 (101.97 to 108.99)
3	702	4.87	144.25 (133.96 to 155.32)	3066	28.13	109.0 (105.21 to 112.93)
4	532	3.73	142.74 (131.11 to 155.40)	2511	21.67	115.87 (111.43 to 120.49)
5 (most deprived)	340	2.18	156.04 (140.30 to 173.54)	1547	14.24	108.67 (103.39 to 114.22)
No records	270			1208		
**Urban-rural 1**						
1 = Urban >10k-Sparce	4	0.02	197.17 (74.0 to 525.33)	4	0.19	21.35 (8.01 to 56.88)
2 = Town & Fringe-Sparce	29	0.24	120.48 (83.73 to 173.38)	96	1.16	82.61 (67.63 to 100.90)
3 = Village, Hamlet, & Isolated dwellings - Sparce	19	0.18	105.66 (67.40 to 165.65)	79	0.92	85.72 (68.76 to 106.87)
4 = Urban > 10k – Less sparse	2265	14.84	152.66 (146.50 to 159.07)	9308	81.19	114.65 (112.34 to 117.0)
5 = Town & Fringe – Less sparse.	387	2.49	155.70 (140.94 to 172.01)	1545	13.51	114.39 (108.83 to 120.24)
6 = Village, Hamlet & Isolated dwellings –	230	1.51	152.07 (133.64 to 173.05)	869	13.50	116.48 (108.99 to 124.49)

Less sparse.						
No records	923			3840	7.46	
**Urban-rural 2**						
Urban	2269	14.86	152.72 (146.56 to 159.13)	9312	81.38	114.43 (112.13 to 116.78)
Town	416	2.73	152.59 (138.61 to 167.99)	1641	14.67	111.86 (106.59 to 117.42)
Rural	249	1.69	147.14 (129.95 to 166.60)	948	8.38	113.10 (106.13 to 120.54)
No records	923			3840		
**UK Countries**						
England	2736	17.73	154.28 (148.61 to 160.17)	11,276	97.17	116.04 (113.92 to 118.21)
Northern Ireland	248	1.25	197.99 (174.82 to 224.23)	988	7.76	127.38 (119.68 to 135.58)
Wales	471	4.48	105.02 (95.96 to 114.95)	1893	24.19	78.26 (74.82 to 81.87)
Scotland	402	2.91	137.71 (124.89 to 151.86)	1584	15.32	103.39 (98.42 to 108.61)
**UK Regions**						
East Midlands	46	0.44	103.57 (77.57 to 138.27)	189	2.12	89.01 (77.19 to 102.65)
East of England	254	1.51	168.37 (148.89 to 190.40)	986	8.10	121.72 (114.35 to 129.56)
London	359	2.44	147.22 (132.75 to 163.27)	1507	13.59	110.92 (105.46 to 116.66)
North East	64	0.59	107.86 (84.42 to 137.80)	280	3.08	90.94 (80.89 to 102.25)
North West	367	2.33	157.49 (142.17 to 174.45)	1573	13.24	118.78 (113.06 to 124.80)
Northern Ireland	248	1.25	197.99 (174.82 to 224.23)	988	7.76	127.38 (119.68 to 135.58)
Scotland	471	4.48	105.02 (95.96 to 114.95)	1893	24.19	78.26 (74.82 to 81.87)
South Central	454	2.86	158.58 (144.65 to 173.86)	1925	15.35	125.39 (119.91 to 131.12)
South East Coast	457	3.09	147.88 (134.93 to 162.08)	1898	16.90	112.34 (107.40 to 117.51)
South West	369	2.13	173.54 (156.71 to 192.18)	1431	11.80	121.22 (115.10 to 127.67)
Wales	402	2.92	137.71 (124.89 to 151.86)	1584	15.32	103.39 (98.42 to 108.61)
West Midlands	292	1.97	148.48 (132.39 to 166.53)	1212	10.74	112.84 (106.67 to 119.38)
Yorkshire and Humber	74	0.37	198.38 (157.96 to 249.15)	275	2.25	122.45 (108.80 to 137.81)

Supplemental Table 3: Adjusted hospitalization rates (adjusted for age, gender, calendar year, social deprivation and smoking) following PD diagnosis/index date for non-PD cohort

**Variables**	**PD cohort**				**Non-PD cohort**						
	Events	Person -Years (1000)	*Adjusted hospitalization rate (95% CI)	p-value	Events	Person-Years (1000)	*Adjusted hospitalization rate (95% CI)	p-value	Incidence rate ratio	**p-value	***p-value
**Years following diagnosis/index date**											
First year	1220	8.75	132.94 (124.74 to 141.14)	<0.001	5312	46.49	109.91 (105.64 to 114.16)	<0.001	1.21 (1.14 to 1.29)	<0.001	0.001
Second year	849	6.28	132.63 (122.96 to 142.30)		3424	32.70	104.05 (99.20 to 108.90)		1.27 (1.19 to 1.37)	<0.001	
Third year	615	4.36	142.58 (129.97 to 155.19)		2436	23.09	107.48 (101.82 to 113.13)		1.32 (1.21 to 1.45)	<0.001	
Fourth year	455	2.89	163.81 (146.50 to 181.12)		1613	15.90	105.43 (98.62 to 112.24)		1.55 (1.40 to 1.72)	<0.001	
Fifth year	319	1.83	187.78 (166.50 to 209.07)		1183	10.69	117.03 (108.50 to 125.57)		1.60 (1.42 to 1.81)	<0.001	
Sixth year	193	1.20	195.84 (167.35 to 224.32)		741	6.88	116.06 (105.52 to 126.60)		1.69 (1.45 to 1.97)	<0.001	
Seventh year	104	0.62	193.04 (154.28 to 231.80)		501	4.22	131.08 (116.72 to 145.44)		1.47 (1.19 to 1.82)	<0.001	
Eighth year	102	0.54	226.55 (183.02 to 270.07)		531	4.46	135.79 (121.58 to 150.00)		1.67 (1.35 to 2.06)	<0.001	

PD-Parkinson's disease

Supplementary Table 4: Crude incidence rates of hospital admissions among male and female people with PD

	Male Parkinson's disease cohort			Female Parkinson's disease cohort		
Variables	Events	Person-Years (1000)	Incidence rate (95% CI)	Events	Person-Years (1000)	Incidence rate (95% CI)
**Overall**	2,343	15.89	147.48 (141.63 to 153.58)	1,514	10.50	144.14 (137.06 to 151.58)
**Age group**						
50 to 59	116	1.08	107.14 (89.31 to 128.52)	67	0.62	107.71 (84.78 to 136.86)
60 to 69	446	3.82	116.79 (106.44 to 128.15)	246	2.13	115.65 (102.06 to 131.04)
70 to 79	951	6.61	143.98 (135.11 to 153.42)	619	4.42	139.91 (129.31 to 151.38)
80 t0 89	753	4.04	186.12 (173.29 to 199.90)	520	2.97	175.09 (160.67 to 190.80)
>90	77	0.33	230.57 (184.41 to 288.27)	62	0.35	175.20 (136.59 to 224.71)
**Townsend quintile**						
1 (least deprived)	707	4.74	149.31 (138.70 to 160.73)	382	2.84	134.73 (121.87 to 148.94)
2	562	3.83	146.84 (135.19 to 159.50)	362	2.33	155.18 (139.99 to 172.02)
3	420	2.93	143.32 (130.25 to 157.70)	282	1.94	145.66 (129.61 to 163.69)
4	294	2.07	142.10 (126.76 to 159.31)	238	1.66	143.54 (126.41 to 162.99)
5 (most deprived)	204	1.22	167.11 (145.68 to 191.69)	136	0.96	141.93 (119.97 to 167.90)
No records	156			114		
**Urban-rural 2**						
Urban	1,365	8.80	155.13 (147.11 to 163.58)	904	6.06	149.22 (139.80 to 159.27)
Town	259	1.72	150.22 (132.99 to 169.67)	157	1.01	156.68 (134.00 to 183.21)
Rural	157	1.69	149.49 (127.84 to 174.80)	92	8.38	143.29 (116.81 to 175.78)
No records	562			361		
**UK Countries**						
England	1,649	10.54	156.40 (149.03 to 164.14)	1,087	7.19	151.17 (142.44 to 118.21)
Northern Ireland	148	0.79	187.00 (159.16 to 219.68)	100	0.46	216.88 (178.28 to 263.84)
Wales	285	2.68	106.41 (94.75 to 119.51)	186	1.81	102.97 (89.18 to 118.88)
Scotland	261	1.87	139.31 (123.40 to 157.28)	141	1.05	134.85 (114.33 to 159.05)

Supplemental Table 5: Adjusted incidence rates and ratios of hospitalization by age group, gender, social deprivation, urban-rural, UK countries.

Variables	PD group				Non-PD control group						
	Events	Person -Years (1000)	*Adjusted Incidence rate (95% CI)	p-value	Events	Person -Years (1000)	*Adjusted Incidence rate (95% CI)	p-value	Incidence rate ratio	**p-value	***p-value
**Age group**											
50 to 59	183	1.70	108.95 (92.39 to 125.50)	<0.001	574	9.49	60.28 (54.70 to 65.86)	<0.001	1.79 (1.52 to 2.11)	<0.001	<0.001
60 to 69	692	5.95	117.82 (108.22 to 127.42)		2546	32.30	79.03 (74.93 to 83.14)		1.49 (1.37 to 1.61)	<0.001	
70 to 79	1570	11.03	144.10 (135.99 to 152.22)		6195	59.39	104.91 (100.63 to 109.20)		1.37 (1.30 to 1.44)	<0.001	
80 to 89	1273	7.02	184.76 (173.64 to 195.87)		5680	39.22	147.86 (141.63 to 154.09)		1.25 (1.17 to 1.32)	<0.001	
>90	139	0.69	204.09 (171.07 to 237.11)		746	4.04	192.59 (175.60 to 209.57)		1.06 (0.89 to 1.26)	0.492	
**Gender**											
Male	2343	15.89	150.21 (142.78 to 157.64)	0.3706	9533	86.54	112.39 (108.15 to 116.63)	0.0457	1.34 (1.29 to 1.40)	<0.001	0.938
Female	1514	10.50	147.21 (138.70 to 155.72)		6208	57.89	108.31 (103.82 to 112.80)		1.35 (1.28 to 1.42)	<0.001	
**Townsend quintile**											
1(least deprived)	1089	7.57	144.33 (133.44 to 155.22)	0.6200	3938	37.03	107.16 (101.72 to 112.61)	0.0110	1.35 (1.27 to 1.44)	<0.001	0.076
2	924	6.16	154. 59 (143.78 to 165.40)		3471	32.03	107.33 (102.17 to 112.49)		1.43 (1.33 to 1.54)	<0.001	
3	702	4.87	148.03 (136.43 to 159.63)		3066	28.13	110.90 (105.13 to 116.67)		1.34 (1.24 to 1.45)	<0.001	
4	532	3.73	146.06 (133.32 to 158.79)		2511	21.67	117.0 (110.97 to 123.02)		1.25 (1.14 to 1.36)	<0.001	
5(most deprived)	340	2.18	156.18 (139.42 to 172.94)		1547	14.24	110.62 (103.55 to 117.69)		1.41 (1.27 to 1.57)	<0.001	
No records	270				1208						
**Urban-rural**											
Urban	2269	14.86	149.45 (137.57 to 161.33)	<0.001	9312	81.38	111.31 (105.31 to 117.31)	0.8632	1.34 (1.28 to 1.41)	<0.001	0.952
Town	416	2.73	149.31 (131.76 to 166.87)		1641	14.67	110.11 (101.05 to 119.18)		1.36 (1.24 to 1.49)	<0.001	
Rural	249	1.69	147.45 (126.79 to 168.10)		948	8.38	114.42 (104.22 to 124.61)		1.31 (1.16 to 1.48)	<0.001	
No records	923				3840						
**UK Countries**											
England	2736	17.73	155.22 (144.46 to 165.98)	<0.001	11,276	97.17	115.90 (110.50 to 121.31)	<0.001	1.33 (1.28 to 1.39)	<0.001	0.141
Northern Ireland	248	1.25	211.0 (159.79 to 262.20)		988	7.76	140.92 (117.53 to 164.30)		1.55 (1.37 to 1.74)	<0.001	

Wales	471	4.48	107.23 (81.70 to 132.75)	1893	24.19	81.69 (67.14 to 96.23)	1.34 (1.23 to 1.45)	<0.001
Scotland	402	2.92	142.06 (127.0 to 157.13)	1584	15.32	106.04 (97.16 to 114.92)	1.33 (1.22 to 1.45)	<0.001

PD Parkinson's disease. *Mutually adjusted for age, gender, calendar year, social deprivation, urban-rural, UK countries and smoking. **Wald test for categorical variables. ***Wald test for interaction terms.

Supplemental Table 6: Adjusted incidence rates and ratios of hospitalization among the PD cohort stratified by gender (adjusted for age group, calendar year, social deprivation, urban-rural, UK countries and smoking).

**Variables**	**PD Male group**				**PD Female group**						
	Events	Person -Years (1000)	*Adjusted Incidence rate (95% CI)	p-value	Events	Person -Years (1000)	*Adjusted Incidence rate (95% CI)	p-value	Incidence rate ratio	**p-value	***p-value
**Overall**	2,343	15.89	150.01 (142.49 to 157.53)	<0.001	1,514	10.50	145.62 (137.16 to 154.07)	<0.001	1.03 (0.72 to 1.14)	0.372	0.984
**Age group**											
50 to 59	116	1.08	107.51 (86.79 to 128.23)	<0.001	67	0.62	107.51 (86.79 to 128.23)	<0.001	1.00 (0.02 to 1.11)	0.060	0.702
60 to 69	446	3.82	117.40 (105.67 to 129.13)		246	2.13	115.91 (101.06 to 130.76)		1.01 (0.68 to 1.44)	0.960	
70 to 79	951	6.61	144.09 (134.28 to 153.92)		619	4.42	142.69 (130.83 to 154.56)		1.00 (0.71 to 1.40)	0.970	
80 to 89	753	4.04	186.80 (173.40 to 200.20)		520	2.97	179.97 (163.84 to 196.08)		1.04 (0.68 to 1.37)	0.847	
>90	77	0.33	228.43 (178.40 to 278.45)		62	0.35	175.92 (134.26 to 217.59)		1.29 (0.49 to 1.38)	0.263	
**Townsend quintile**											
1(least deprived)	707	4.74	145.21 (133.65 to 156.77)	0.6200	382	2.84	140.96 (129.35 to 152.57)	0.0110	1.05 (1.07 to 1.14)	0.078	0.422
2	562	3.83	155. 57 (144.21 to 166.93)		362	2.33	151.02 (138.77 to 163.28)		1.16 (0.99 to 1.38)	0.068	
3	420	2.93	149.45 (131.94 to 158.21)		282	1.94	145.08 (131.94 to 158.21)		1.10 (0.92 to 1.31)	0.300	
4	294	2.07	147.31 (134.02 to 160.59)		238	1.66	143.00 (129.33 to 156.67)		1.13 (0.76 to 1.25)	0.246	
5(most deprived)	204	1.22	157.63 (140.13 to 175.13)		136	0.96	153.02 (135.96 to 170.08)		1.01 (0.76 to 1.55)	0.825	
No records	156				114						
**Urban-rural**											
Urban	1,365	8.80	150.53 (138.01 to 163.05)	<0.001	904	6.06	146.13 (133.59 to 158.66)	0.8632	1.03 (0.83 to 1.13)	0.728	0.957
Town	259	1.72	149.94 (132.02 to 167.86)		157	1.01	145.55 (127.84 to 163.26)		1.02 (0.81 to 1.30)	0.844	
Rural	157	1.69	148.26 (127.44 to 169.07)		92	8.38	143.92 (123.01 to 164.83)		1.03 (0.72 to 1.27)	0.790	
No records	562				361						
**UK Countries**											
England	1,649	10.54	156.48 (145.24 to 167.71)	<0.001	1,087	7.19	151.90 (139.88 to 163.90)	<0.001	1.03 (0.83 to 1.14)	0.234	0.644
Northern Ireland	148	0.79	212.17 (160.13 to 264.20)		100	0.46	205.95 (155.44 to 256.47)		1.22 (0.89 to 1.65)	0.215	
Wales	285	2.68	108.75 (80.24 to 130.90)		186	1.81	105.57 (80.24 to 130.90)		1.03 (0.80 to 1.19)	0.837	
Scotland	261	1.87	143.70 (128.03 to 159.36)		141	1.05	139.49 (123.48 to 155.50)		1.03 (0.81 to 1.21)	0.905	

PD-Parkinson's disease. *Mutually adjusted for age, calendar year, social deprivation, urban-rural, UK countries and smoking. **Wald test for categorical variables. ***Wald test for interaction terms.

Supplemental Table 7: Reasons for hospitalization among the admitted cohort who are in the younger age group (50 to 59 and 60 to 69 years).

	**PD cohort**		**Non-PD control cohort**			
Reasons for hospital admission	Number admitted	Rate admitted for the reason per 1,000 person-years (95% Confidence Interval)	Number admitted	Rate admitted for the reason per 1,000 person-years (95% Confidence Interval)	Incidence rate ratio (95% Confidence Interval)	*p-value
Neuropsychiatric complications (psychosis and hallucinations)	45	4.61 (3.44 to 6.18)	12	0.23 (0.13 to 0.40)	19.46 (10.65 to 35.56)	<0.001
Dementia	102	10.45 (8.61 to 12.69)	48	0.92 (0.69 to 1.22)	11.41 (7.96 to 16.35)	<0.001
Myocardial infarction/Ischaemic heart disease	30	3.07 (2.15 to 4.40)	157	3.00 (2.56 to 3.50)	0.96 (0.65 to 1.43)	0.851
Congestive heart failure	19	1.95 (1.24 to 3.05)	102	1.95 (1.60 to 2.36)	1.00 (0.60 to 1.65)	0.986
Stroke	31	3.18 (2.23 to 4.52)	132	2.50 (2.10 to 2.97)	1.28 (0.84 to 1.93)	0.245
Hypertension	42	4.30 (3.18 to 5.83)	226	4.31 (3.79 to 4.91)	0.94 (0.68 to 1.32)	0.733
Gastrointestinal complications (dysphagia, constipation, nausea and vomiting)	140	14.35 (12.16 to 16.93)	285	5.44 (4.84 to 6.11)	2.57 (2.07 to 3.21)	<0.001
Falls	109	11.17 (9.26 to 13.48)	174	3.32 (2.86 to 3.85)	3.28 (2.54 to 4.24)	<0.001
Fractures	89	9.02 (7.32 to 11.12)	139	2.60 (2.19 to 3.07)	3.32 (2.52 to 4.37)	<0.001
Infections	125	12.81 (10.75 to 15.27)	386	7.35 (6.65 to 8.12)	1.74 (1.41 to 2.13)	<0.001
Recorded as cardiovascular causes	56	5.74 (4.42 to 7.46)	273	5.21 (4.63 to 5.87)	1.06 (0.78 to 1.43)	0.711
Cancer	78	7.99 (6.40 to 9.98)	369	7.04 (6.36 to 7.80)	1.10 (0.86 to 1.41)	0.449
Postural hypotension	67	6.87 (5.41 to 8.73)	56	1.05 (0.81 to 1.37)	6.32 (4.36 to 9.15)	<0.001
Electrolyte imbalance	21	2.15 (1.40 to 3.30)	49	0.94 (0.71 to 1.24)	2.31 (1.39 to 3.84)	0.001
Parkinson's disease	44	4.00 (2.92 to 5.47)	NA	NA	NA	NA
Surgical causes	85	8.71(7.04 to 10.78)	320	6.11 (5.48 to 6.82)	1.37 (1.05 to 1.78)	0.018
Not identified	75		1,368			

PD-Parkinson's disease. *Mutually adjusted for age, gender, calendar year, social deprivation and smoking.

Supplemental Table 8: Reasons for hospitalization among the admitted cohort who are in the older age group (70 years and more).

	Parkinson's disease cohort		Non-Parkinson's disease control cohort			
Reasons for hospital admission	Number admitted	Rate admitted for the reason per 1,000 person-years (95% Confidence Interval)	Number admitted	Rate admitted for the reason per 1,000 person-years (95% Confidence Interval)	Incidence rate ratio (95% Confidence Interval)	*p-value
Neuropsychiatric complications (psychosis and hallucinations)	93	5.59 (4.56 to 6.85)	85	0.92 (0.75 to 1.14)	5.91 (4.37 to 8.01)	<0.001
Dementia	180	18.82 (16.84 to 21.02)	620	6.54 (6.04 to 7.08)	2.83 (2.45 to 3.28)	<0.001
Myocardial infarction/Ischaemic heart disease	56	3.37 (2.59 to 4.37)	412	4.41 (4.00 to 4.86)	0.74 (0.56 to 1.00)	0.036
Congestive heart failure	76	4.57 (3.65 to 5.72)	547	5.93 (5.45 to 6.45)	0.76 (0.59 to 1.00)	0.038
Stroke	137	8.18 (6.91 to 9.67)	566	6.12 (5.63 to 6.64)	1.31 (1.10 to 1.57)	0.003
Hypertension	50	3.01 (2.28 to 3.97)	413	4.40 (4.00 to 4.85)	0.68 (0.50 to 0.93)	0.015
Gastrointestinal complications (dysphagia, constipation, nausea and vomiting)	277	16.65 (14.80 to 18.73)	1,045	11.35 (10.68 to 12.06)	1.45 (1.27 to 1.66)	<0.001
Falls	408	24.53 (22.26 to 27.03)	1,032	11.21 (10.55 to 11.92)	2.17 (1.94 to 2.44)	<0.001
Fractures	227	13.65 (11.98 to 15.54)	595	6.41 (5.91 to 6.95)	2.11 (1.79 to 2.48)	<0.001
Infections	319	19.18 (17.18 to 21.40)	1,237	13.44 (12.71 to 14.21)	1.42 (1.26 to 1.61)	<0.001
Recorded as cardiovascular causes	156	9.32 (7.96 to 10.91)	779	8.39 (7.82 to 9.00)	1.09 (0.91 to 1.30)	0.338
Cancer	172	10.34 (8.90 to 12.01)	1,037	11.20 (10.54 to 11.90)	0.92 (0.78 to 1.08)	0.312
Postural hypotension	162	9.74 (8.35 to 11.36)	285	3.07 (2.74 to 3.45)	3.08 (2.54 to 3.73)	<0.001
Electrolyte imbalance	58	3.49 (2.70 to 4.51)	246	2.67 (2.36 to 3.03)	1.31 (0.97 to 1.76)	0.078
Parkinson's disease	133	8.30 (7.02 to 9.80)	NA	NA	NA	NA
Surgical causes	188	11.24 (9.74 to 12.97)	787	8.54 (7.96 to 9.16)	1.27 (1.09 to 1.49)	0.003
Not identified	7		1,959			

PD-Parkinson's disease. *Mutually adjusted for age, gender, calendar year, social deprivation and smoking.

Supplemental Table 9: Reasons for hospitalization among the admitted PD cohort stratified by gender

		Male PD cohort		Female PD control cohort			
Reasons for hospital admission	Number admitted within the PD cohort		Rate admitted for the reason per 1,000 person-years (95% Confidence Interval)	Number admitted	Rate admitted for the reason per 1,000 person-years (95% Confidence Interval)	Incidence rate ratio (95% Confidence Interval)	*p-value
Neuropsychiatric complications (psychosis and hallucinations)	138	83	5.22 (4.21 to 6.48)	55	5.24 (4.02 to 6.82)	1.04 (0.73 to 1.48)	0.814
Myocardial infarction/Ischaemic heart disease	86	65	4.09 (3.21 to 5.22)	21	2.08 (1.27 to 3.40)	2.08 (1.27 to 3.40)	0.003
Congestive heart failure	95	61	3.84 (2.99 to 4.94)	34	3.24 (2.31 to 4.53)	1.30 (0.83 to 2.05)	0.255
Stroke	168	94	5.92 (4.83 to 7.24)	74	6.95 (5.53 to 8.74)	0.92 (0.69 to 1.23)	0.578
Hypertension	92	59	3.71 (2.88 to 4.79)	33	3.14 (2.23 to 4.42)	1.21 (0.75 to 1.95)	0.428
Gastrointestinal complications	417	251	15.80 (13.96 to 17.88)	166	15.80 (13.57 to 18.40)	1.02 (0.82 to 1.24)	0.883
Falls	517	328	20.65 (18.53 to 23.01)	189	18.00 (15.60 to 20.75)	1.20 (1.01 to 1.44)	0.040
Fractures	316	151	9.44 (8.05 to 11.08)	165	15.02 (13.49 to 18.30)	0.62 (0.50 to 0.78)	<0.001
Infections	444	284	17.88 (15.91 to 20.08)	160	15.23 (13.05 to 17.79)	1.22 (1.01 to 1.48)	0.036
Other cardiovascular causes	212	132	8.31(7.01 to 9.85)	80	7.52 (6.03 to 9.38)	1.16 (0.86 to 1.52	0.304
Cancer	250	156	9.82 (8.39 to 11.49)	94	8.95 (7.31 to 10.95)	1.11 (0.85 to 1.45)	0.447
Postural hypotension	229	153	9.63 (8.23 to 11.28)	76	7.24 (5.78 to 9.06)	1.40 (1.07 to 1.84)	0.015
Electrolyte imbalance	79	39	2.45 (1.79 to 3.36)	40	3.81 (2.79 to 5.19)	0.67 (0.42 to 1.05)	0.078
Parkinson's disease	177	110	6.92 (5.74 to 8.35)	67	6.38 (5.02 to 8.10)	1.13 (0.82 to 1.55)	0.442
Surgical causes	273	160	10.00 (8.57 to 11.69)	113	10.76 (8.95 to 12.94)	0.93 (0.74 to 1.18)	0.559
Not identified	217			147			

PD-Parkinson's disease. *Mutually adjusted for age, gender, calendar year, social deprivation and smoking.

Supplemental Table 10: Reasons for hospitalization among the admitted cohort (Unadjusted IRRs)

	**Parkinson's disease cohort**			**Non-Parkinson's disease control cohort**				
Reasons for hospital admission	Number admitted	%	Rate admitted for the reason per 1,000 person-years (95% Confidence Interval)	Number admitted	%	Rate admitted for the reason per 1,000 person-years (95% Confidence Interval)	Incidence rate ratio (95% Confidence Interval)	*p-value
Neuropsychiatric complications (psychosis and hallucinations)	138	3.58	5.23 (4.43 to 6.18)	97	0.62	0.67 (0.55 to 0.82)	7.79 (6.01 to 10.10)	<0.001
Dementia	282	4.15	15.73 (14.28 to 17.31)	688	1.18	4.50 (4.17 to 4.86)	3.49 (3.06 to 3.99)	<0.001
Myocardial infarction/Ischaemic heart disease	86	2.23	3.26 (2.64 to 4.03)	569	3.61	3.90 (3.59 to 4.23)	0.84 (0.67 to 1.04)	0.112
Congestive heart failure	95	2.46	3.60 (2.94 to 4.40)	649	4.12	3.60 (2.94 to 4.40)	0.80 (0.64 to 1.01)	0.056
Stroke	168	4.36	6.33 (5.44 to 7.36)	698	4.43	4.80 (4.46 to 5.18)	1.32 (1.12 to 1.54)	0.001
Hypertension	92	2.39	3.49 (2.84 to 4.28)	639	4.06	4.37 (4.04 to 4.72)	0.80 (0.64 to 1.0)	0.051
Gastrointestinal complications	417	10.81	15.80 (14.36 to 17.39)	1,330	8.45	9.21 (8.73 to 9.72)	1.72 (1.53 to 1.92)	<0.001
Falls	517	13.40	19.59 (17.97 to 21.35)	1,206	7.66	8.35 (7.89 to 8.83)	2.35 (2.12 to 2.60)	<0.001
Fractures	316	8.19	11.94 (10.69 to 13.33)	734	4.66	5.02 (4.67 to 5.41)	2.37 (2.07 to 2.72)	<0.001
Infections	444	11.51	16.82 (15.33 to 18.46)	1,623	10.31	11.23 (10.70 to 11.79)	1.50 (1.35 to 1.67)	<0.001
Recorded as cardiovascular causes	212	5.50	8.0 (6.99 to 9.15)	1,052	6.68	7.23 (6.81 to 7.69)	1.10 (0.95 to 1.28)	0.193
Cancer	250	6.48	9.47 (8.37 to 10.72)	1406	8.93	9.69 (9.20 to 10.21)	0.98 (0.85 to 1.12)	0.738
Postural hypotension	229	2.29	8.68 (7.62 to 9.88)	341	0.61	2.34 (2.10 to 2.60)	3.70 (3.14 to 4.37)	<0.001
Electrolyte imbalance	79	0.79	2.99 (2.40 to 3.73)	295	0.53	2.04 (1.82 to 2.29)	1.47 (1.14 to 1.88)	0.003
Parkinson's disease	177	4.59		NA	NA	NA	NA	NA
Surgical causes	273	2.73	10.31 (9.15 to 11.61)	1,107	1.99	7.66 (7.22 to 8.12)	1.35 (1.17 to 1.54)	<0.001
Not identified								

***unadjusted**
